# Supplementary material for: The crucial role of circular waste management systems in cutting waste leakage into aquatic environments
Source: Nat Commun. 2024 Jun 27;15:5443. doi: 10.1038/s41467-024-49555-9 (PMC11211435; doi:10.1038/s41467-024-49555-9)
Supplement: Supplementary file 1 — Supplementary Information [file 41467_2024_49555_MOESM1_ESM.pdf]

Supplementary Information to :

The crucial role of circular waste management systems in cutting waste leakage into aquatic environments

Adriana Gómez-Sanabria [1*] & Florian Lindl[1]

[1] Pollution Management Research Group. Energy, Climate and Environment Program, International Institute for Applied Systems Analysis, Laxenburg, Austria

Corresponding author

Correspondence to: Adriana Gómez-Sanabria. Email: gomezsa@iiasa.ac.at

Table of Contents

S1 Composition of scattered MSW by region 2

S2. Scattered MSW by region 4

S3. Studies assessing waste leakage into aquatic environments 5

S4. Top 20 countries with the highest MSW 7

S5. Summary of the methodology to estimate MSW generation and composition 9

S6. Narratives of mitigation scenarios 13

S7. Development of MSW management by mitigation scenario and fraction of scattered MSW by stream 19

S8. GAINS country/region and regional aggregation 22

S1 Composition of scattered MSW by region

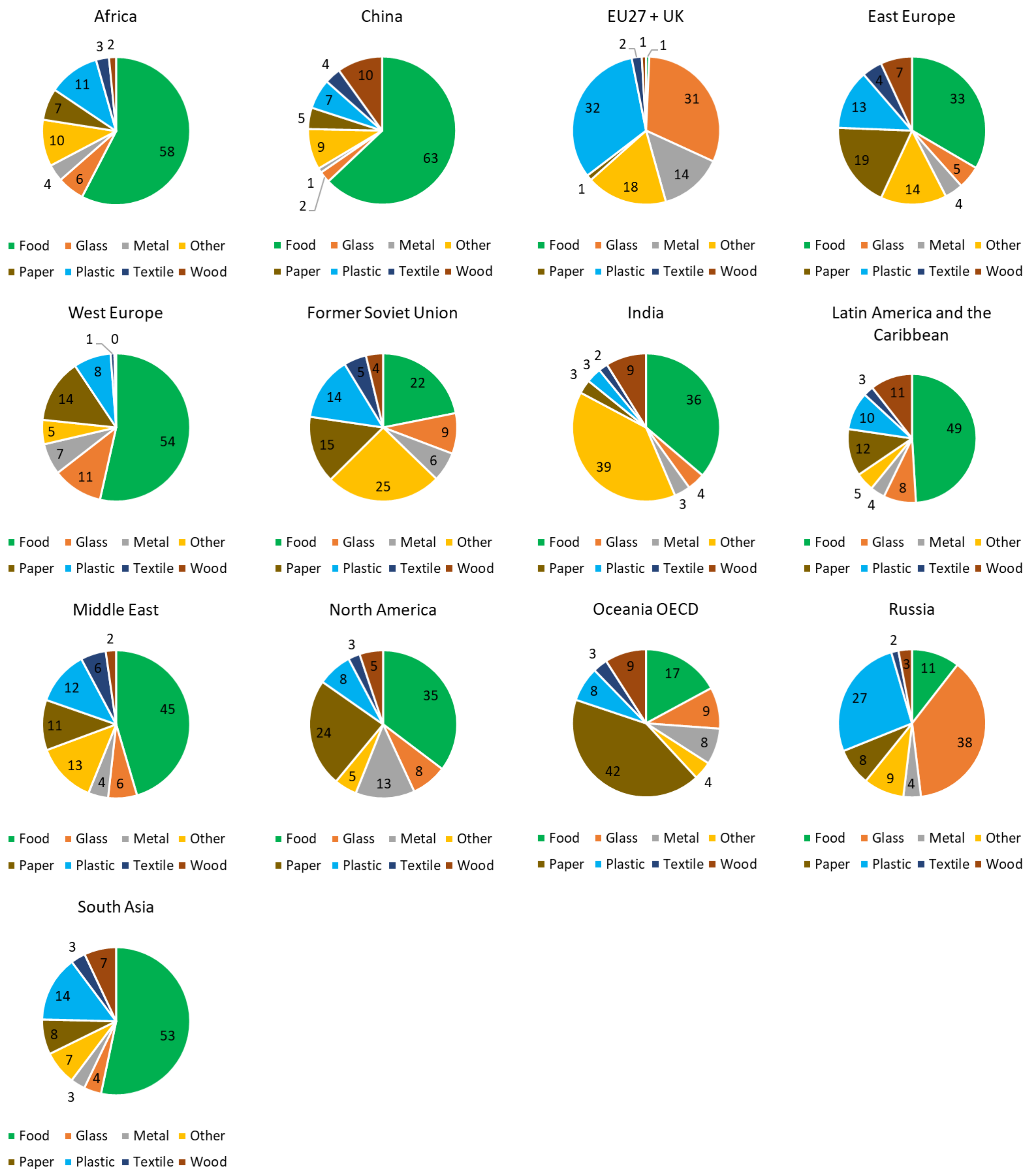

Fig S1. Composition of scattered MSW by region in 2020

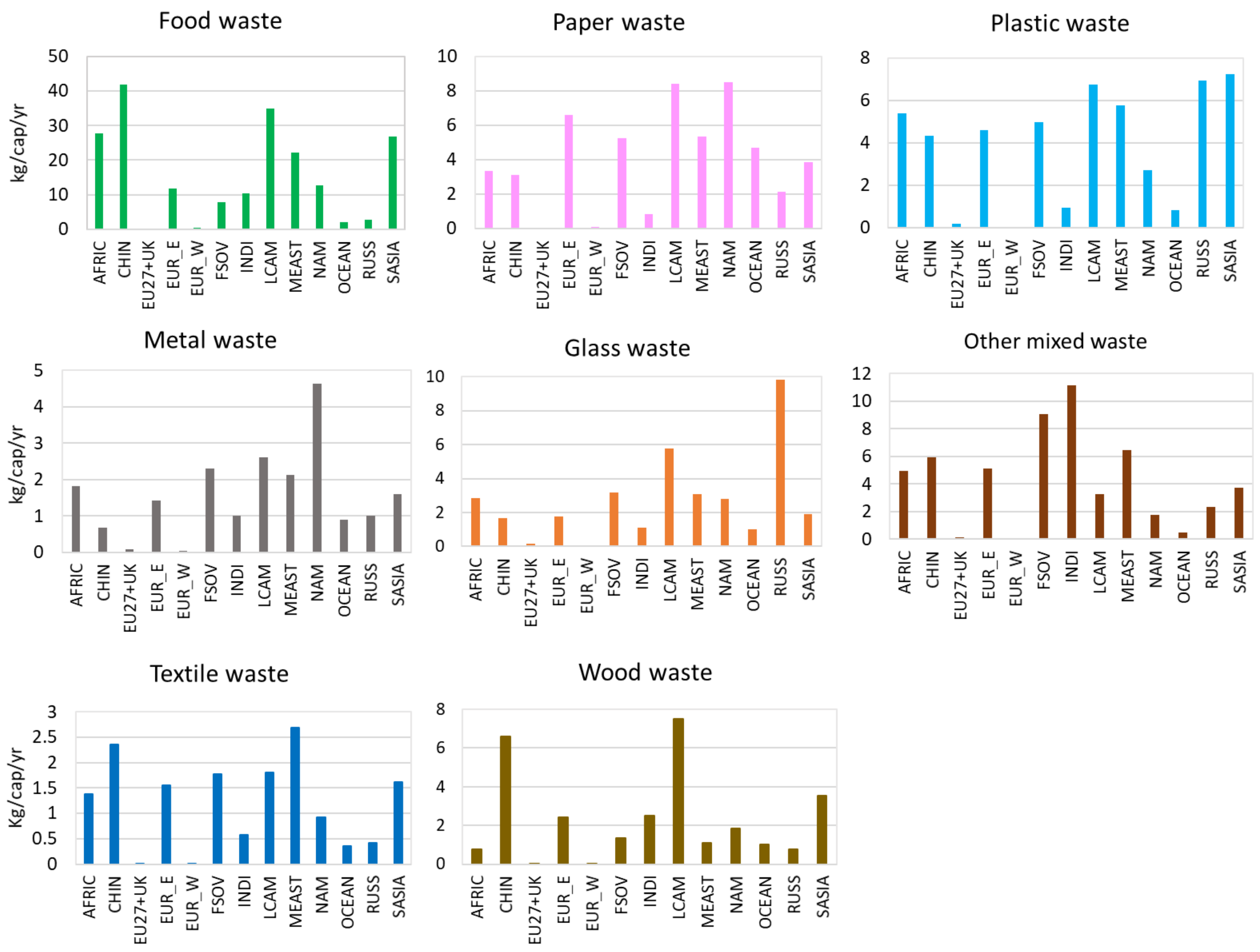

Fig S2. Composition of scattered MSW by region in 2020 in kg/cap/yr

S2. Scattered MSW by region

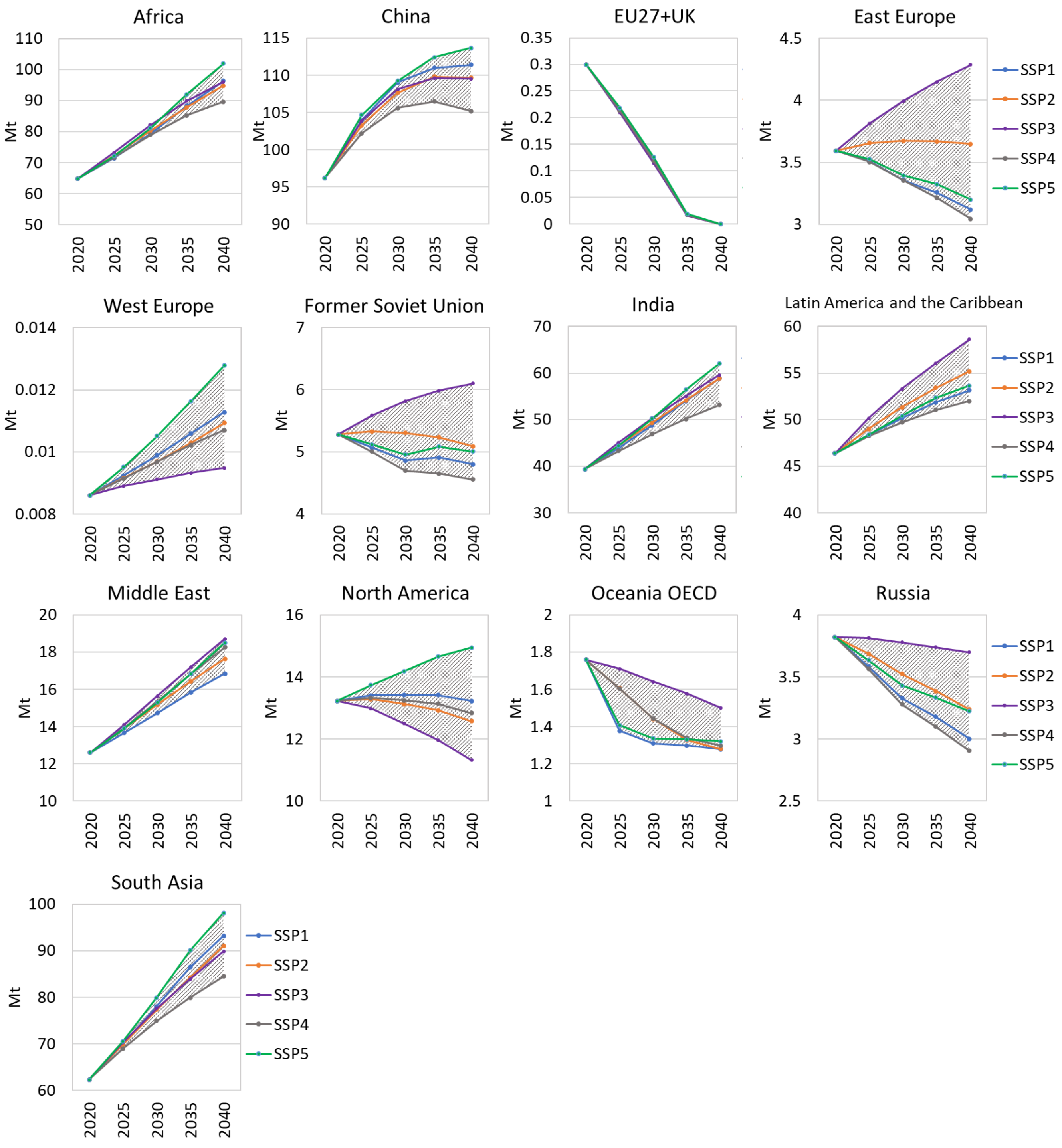

Fig S3. Scattered MSW by region and SSP in the *Baseline* scenario

Table S1. Municipal solid waste generation and scattered waste

		Global municipal solid waste generation in Mt					Global Municipal solid waste leakage in Mt				
**Scenario**	**CI 95%**	**2020**	**2025**	**2030**	**2035**	**2040**	**2020**	**2025**	**2030**	**2035**	**2040**
SSP1_Baseline	2.50%	2560	2759	2946	3121	3271	349	370	388	406	418
	Estimate	2560	2822	3082	3335	3563	349	378	406	433	455
	97.50%	2560	2925	3309	3698	4062	349	392	436	481	519
SSP2_Baseline	2.50%	2560	2758	2937	3097	3241	349	373	394	412	426
	Estimate	2560	2810	3042	3253	3448	349	380	408	432	453
	97.50%	2560	2896	3216	3515	3798	349	392	432	467	499
SSP3_Baseline	2.50%	2560	2755	2923	3064	3185	349	378	403	424	440
	Estimate	2560	2801	3007	3177	3322	349	385	415	439	459
	97.50%	2560	2875	3143	3362	3547	349	395	434	465	490
SSP4_Baseline	2.50%	2560	2748	2915	3058	3173	349	368	384	395	402
	Estimate	2560	2800	3020	3210	3369	349	375	397	415	427
	97.50%	2560	2883	3187	3455	3683	349	386	419	447	467
SSP5_Baseline	2.50%	2560	2781	3000	3220	3412	349	372	392	414	429
	Estimate	2560	2854	3161	3487	3785	349	381	413	448	476
	97.50%	2560	2971	3424	3930	4407	349	397	448	505	554
		**Scattered MSW in urban in Mt**					**Scattered MSW in urban in Mt**				
SSP1_Baseline	2.50%	242	269	296	319	339	107	101	93	87	79
	Estimate	242	276	309	341	369	107	103	97	93	86
	97.50%	242	286	332	378	420	107	107	104	103	98
SSP2_Baseline	2.50%	242	267	291	311	330	107	106	104	101	96
	Estimate	242	272	301	327	350	107	108	107	106	103
	97.50%	242	281	318	353	386	107	112	113	114	113
SSP3_Baseline	2.50%	242	267	288	306	322	107	112	115	117	118
	Estimate	242	271	296	317	336	107	114	118	122	123
	97.50%	242	278	310	336	359	107	117	124	129	132
SSP4_Baseline	2.50%	242	268	291	310	325	107	101	93	86	78
	Estimate	242	273	302	325	345	107	103	96	90	82
	97.50%	242	281	318	350	377	107	106	101	97	90
SSP5_Baseline	2.50%	242	271	298	324	346	107	105	99	92	87
	Estimate	242	278	314	351	384	107	107	104	100	97
	97.50%	242	289	340	396	447	107	112	113	112	113

S3. Studies assessing waste leakage into aquatic environments

Table S2. Studies assessing leakage of waste into aquatic environments

Scale	Type	Description	Source
Global	Rivers	1.15 – 2.41 Mt of plastic waste from rivers enters the ocean every year.	(Lebreton et al., 2017)[1]
Global	Rivers	0.8 – 2.7 Mt of plastic waste from rivers enters the ocean. Small urban rivers are the most polluting	(Meijer et al., 2019)[2]

Global	Rivers	60 – 90 Mt of mismanaged plastic waste were produced in 2015 and it is projected to increase up to 155 – 265 Mt/yr by 2060.	(Lebreton et al., 2019)[3]
Global	Rivers	0.41 – 4 Mt of plastic debris inputs from rivers into the sea	(Schmidt et al., 2017)[4]
Global	Coastal areas	Between 4.8 to 12.7 Mt of plastic waste from 192 coastal countries entered the ocean in 2010	(Jambeck., 2015)[5]
Global	Terrestrial and aquatic environments	22 Mt of plastic leaked into the environment. 86% from mismanaged waste in 2019. 6.1 Mt reaching aquatic systems in 2019. Mismanaged plastic waste into the aquatic environment is projected to increase by 91% in 2060.	(Global Plastics Outlook., 2022)[6]
Global	Aquatic ecosystems	Amount of plastic waste entering aquatic ecosystems in 2016 is assessed at 9 -14 Mt per year and it is projected to increase up to 19-23 Mt per year by 2030.	(UNEP, 2021)[7]
Global	Aquatic ecosystems	Evaluates interventions to reduce plastic pollution at municipal level and four sources of microplastics through different scenarios. Interventions will reduce plastic pollution by 40% and 78% relative to "business as usual" in 2040	(Lau et al., 2020)[8]
Global	Aquatic ecosystems	Three scenarios to evaluate mitigation strategies to reduce plastic emissions. 19 to 23 Mt of plastic waste entered aquatic environments in 2016. Annual emissions are projected to increase up to 53 Mt/yr by 2030.	(Borrelle et al., 2020)[9]
Global	Aquatic ecosystems	80.8 Mt of MSW leaked into aquatic environments in 2020 and it's projected to increase up to 22% (SSP4) - 36% (SSP5) in 2040 under the *Baseline* scenarios. Leakage into rivers accounts for 91% of the total MSW reaching aquatic systems. 8.09 Mt of plastic waste reached aquatic ecosystems in 2020. Mismanaged plastic entering aquatic environments is projected to increase up to 9.6 Mt per year by 2030 and by 34% compared to the current level in 2040 if current conditions are maintained.	This study
Ethiopia	Lakes	1.68 t/day of uncollected plastic ends up in Lake Hawassa (Ethiopia)	(RWA., 2020)[10]
United States	Lakes	9.887 kt per year of plastic debris enters the Great Lakes .	(Hoffman et al., 2017)[11]
Eastern Carpathians	Lakes	The study assesses the number of plastic bottles dumped into the Izvoru Muntelui lake. Rural municipalities are responsible for 85.51% of total plastic bottles collected during 2005–2010.	(Mihai-F-C., 2018)[12]
Carpathian	Rivers	The study identifies that watercourses below 750 m.a.s.l are significantly affected by mismanaged plastic waste and most of the hotspots are located in Romania, Hungary, and the Ukraine	(Liro et al., 2023)[13]
Indonesia	Rivers	2603 tons per year of plastic found along the mainstream of the Ciliwung River and 1547 tons per year to the Cikapundung River.	(Rinasti et.,al 2022)[14]

Jakarta	Rivers	2100 tons of plastic waste transported from land to sea per year. This represents 3% of unmanaged plastic waste in Jakarta	(Van Emmerik et.al., 2019)[15]
Europe 27 + UK	Coastal areas	26.1 Mt of small non-packaging plastic items were generated in 2018. 90% of these items in European coastal territories are generated in the Mediterranean and the Black Sea regions.	(Winterstetter et al., 2023)[16]

S4. Leakage of MSW in aquatic environments

Table S3. Coastal areas with the highest potential MSW leakage

MSW (Mt)	GAINS Region	Countries
0.310	CARB_WHOL	Martinique, Puerto Rico, Saint Lucia, Saint Vincent and the Grenadines, Suriname, Trinidad and Tobago, United States Virgin Islands, Anguilla, Antigua and Barbuda, Caribbean Netherlands, Cayman Islands, Curaçao, Dominica, Aruba, Bahamas, Barbados, Cuba, Dominican Republic, French Guiana, Grenada, Guadeloupe, Guyana, Haiti, Jamaica, British Virgin Islands
0.238	WAFR_WHOL	Mali, Mauritania, Niger, Senegal, Sierra Leone, Togo, Benin, Burkina Faso, Cameroon, Cape Verde Central African Republic, Chad, Congo, Cote d'Ivoire, Democratic Republic of the Congo, Equatorial Guinea, Gabon, Gambia, Ghana, Guinea, Guinea-Bissau, Liberia
0.235	NAFR_WHOL	Morocco, Tunisia, Algeria, Libya, Western Sahara
0.146	Myanmar	
0.420	Brazil	
0.148	MIDE_WHOL	State of Palestine, Oman, Qatar, Syrian Arab Republic, United Arab Emirates, Yemen, Bahrain, Iraq, Jordan, Kuwait, Lebanon
0.346	Philippines	
0.785	Indonesia	
0.854	China	
0.284	India	

Table S4. Rivers with the highest potential MSW leakage

MSW in Mt	River name	Basin
5.621	Duke He	Yangtze
2.595	Wei He	Yellow river
1.250	Adar	Nile
1.223	Brahmaputra	Brahmaputra
1.140	Bei	Zhu Jiang
0.972	Daling	Yellow sea and east china sea
0.809	Chenab	Indus
0.742	Bermejo	Rio de la Plata
0.738	Hau (also Basak)	Mekong
0.707	Bagoe	Niger

Table S5. Lakes with the highest potential MSW leakage

MSW in Mt	Lake name	Lake area km^2
0.024	Yuan Jiang	113.79
0.030	Bay	897.36
0.022	Billings Reservoir	99.72
0.052	Ebrie Lagoon	513.55
0.029	Kivu	2400.88
0.025	Poyang	2398.32
0.054	Tanganyika	32826.65
0.036	Three Gorges Reservoir	852.97
0.073	Victoria	67166.22
0.019	Volta	6045.16

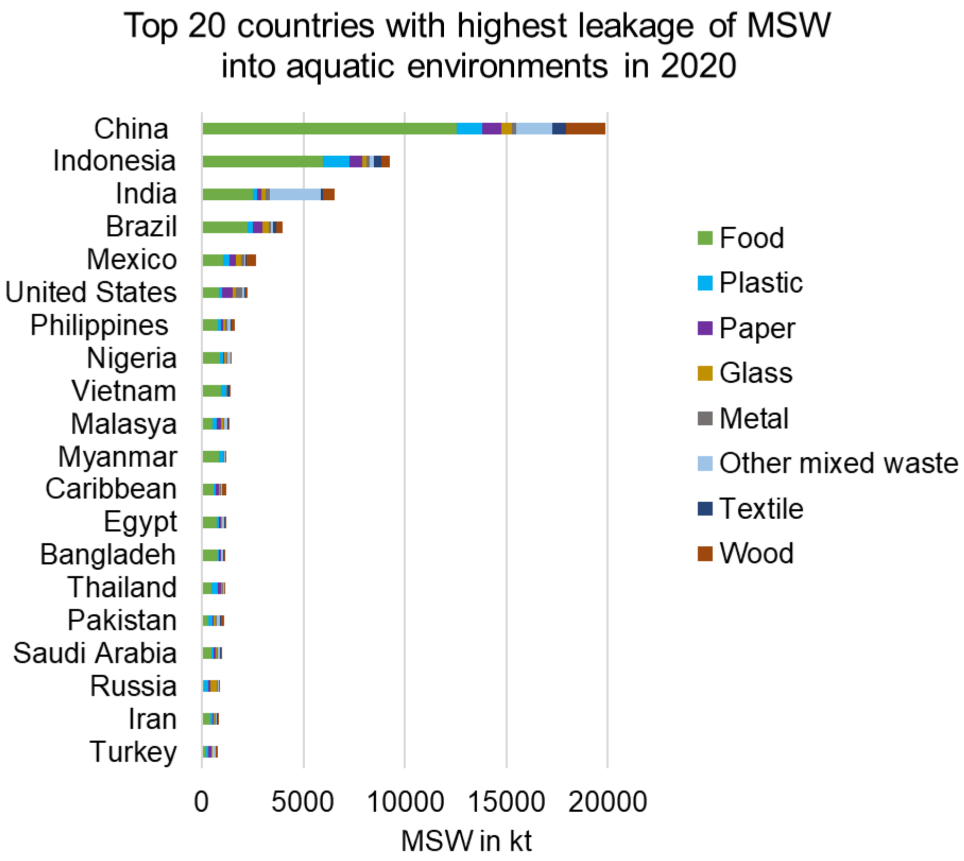

Fig S4. Top 20 countries with highest estimated leakage of MSW into aquatic environments (rivers, lakes, and coastal areas) in 2020. Although food waste accounts for most of leakage, plastic is the material of major concern. Plastic breaks down to form micro and -nanoplastics which threat the aquatic environment and human health.

S5. Summary of the methodology to estimate MSW generation and composition

The definition of MSW used in this study is consistent with that in the ref[17] which is the definition adopted in the Directive 851 of the European Parliament and of the council of 30 May 2018 amending Directive 2008/98/EC on waste[18]: "Municipal waste is defined as waste from households and waste from other sources, such as retail, administration, education, health services, accommodation and food services, and other services and activities, which is similar in nature and composition to waste from households. Therefore, municipal waste includes, inter alia, waste from park and garden maintenance, such as leaves, grass and tree clippings, and waste from market and street cleaning services, such as the content of litter containers and sweepings except materials such as sand, rock, mud or dust".

Corresponding adjustments have been carried out as the definition of MSW generation across countries suffers from inconsistencies thereby introducing higher uncertainties to the estimates.

The approach to estimate MSW generation and composition is adopted from our recent methodology presented ref[17] in which MSW generation projections are based on the assumption that MSW generation and composition depend on the average income level. The method estimates MSW generation per capita elasticities to GDP per capita for different income groups: Low-income group is formed by countries/regions with GDP per capita lower than 9500 US$/year, middle-income group represents countries/regions with GDP per capita higher-equal than 9500 US$/year and lower than 22000 US$/year; middle-high income group represents countries/regions with GDP per capita higher-equal than 22000 US$/year and lower than 38000 US$/year and high-income group is formed by countries/regions with GDP per capita higher equal than 38000 US$/year.

Table S6. MSW generation elasticities to GDP per capita and urbanization rate

Income group USD2010/cap	n	Number of observations	Explanatory variable	OLS	Fixed Effect	Random Effect	LM - test	Hausman - test	CI (95%)	
									Low bounday (2.5%)	high boundary (97.5%)
<9500	23	166	Constant	0.001		0.015	8.835	23.012		
			GDP per capita	0.000	0.375***	0.008				
			Urbanization rate	0.013	0.003	0.011				
			R-square	-0.010	-0.001	-0.008				
<9500	23	166	Constant	-0.001		0.013	8.923	23.643		
			GDP per capita	0.000	**0.375*** **	0.007			0.265	0.588
			Urbanization rate							
			R-square	-0.006	**0.006**	0.446				
<9500	23	166	Constant	0.001		0.010	6.692	0.063		
			GDP per capita							
			Urbanization rate	0.013	0.009	0.011				
			R-square	-0.004	-0.161	-0.004				
>=9500 - <22000	18	253	Constant	-0.024**		-0.023~	2.351	2.438		
			GDP per capita	0.250***	0.175**	0.224***				
			Urbanization rate	-0.504~	-0.127	-0.357				
			R-square	0.080	-0.032	0.060				
>=9500 - <22000	18	253	Constant	-0.046***		**-0.022**	34.273	1.818		
			GDP per capita	0.364***	0.160**	**0.183*** **			0.093	0.27
			Urbanization rate							
			R-square	0.085	-0.028	**0.058**				
>=9500 - <22000	18	253	Constant	-0.016*		-0.015	3.286	1.054		
			GDP per capita							
			Urbanization rate	0.291	0.551*	0.437~				
			R-square	0.002	-0.059	0.008				
>=22000 - <38000	22	201	Constant	-0.011		-0.021	8.378	0.368		
			GDP per capita	0.204***	0.258**	0.241***				
			Urbanization rate	-0.263	-0.042	-0.104				
			R-square	0.096	-0.061	0.078				
>=22000 - <38000	22	201	Constant	-0.011		**-0.021**	8.616	0.174		
			GDP per capita	0.186***	0.254***	**0.233*** **			0.083	0.306
			Urbanization rate							
			R-square	0.090	-0.055	**0.081**				
>=22000 - <38000	22	201	Constant	-0.007		-0.022	7.436	9.389		
			GDP per capita							
			Urbanization rate	-0.027	0.312	0.088				
			R-square	-0.005	-0.115	0.006				
>=38000*	16	230	Constant	-0.001		-0.001	0.051	0.024		
			GDP per capita	0.536***	0.537 ***	0.536***				
			Urbanization rate	0.027	0.019	0.027				
			R-square	0.8971	0.256	0.303				
>=38000*	16	230	Constant	-0.001		-0.001	0.051	0.002		
			GDP per capita	0.539***	0.539***	**0.539*** **			0.342	0.738
			Urbanization rate							
			R-square	0.307	0.259	**0.307**				
>=38000*	16	230	Constant	-0.003		-0.003	0.043	0.068		
			GDP per capita							
			Urbanization rate	0.473**	0.488**	0.473**				
			R-square	0.027	-0.041	0.027				

Where:, $\varepsilon_{it}=u_i+v_{it}$ is an error term which is separated into an individual effects term and a residual omitted variables term, and ε_{it}~IID $\left(0, \sigma_{\varepsilon}^{2}\right)$ is an error term which are assumed to be normally distributed with mean zero and constant variance. Fixed model one-sided and random model two-sided. <9500 one-way (individual) effect within model, F-statistic: 17.2095, p-value: 0.000058878, >=9500 - <22000 ne-way (individual) effect within model Chisq: 16.2488, p-value: 0.000055543, >=22000 - <38000 one-way (individual) random effect within model Chisq: 11.7156, p-value: 0.00061978, >=38000 one-way (individual) random effect within model Chisq: 19.8564, p-value:

0.0000083484 **.** ** before implementation of waste prevention programs or policies to reduce MSW generation.* Source: Gómez-Sanabria et al., 2022[17].

S6. Narratives of mitigation scenarios

Our scenarios adopt the socio-economic narratives from the Shared Socioeconomic Pathways (SSPs) (see O'Neill et al, 2017[19]). The SSPs provide five plausible pathways about probable world's socioeconomic development. Based on our interpretation of the narratives, we develop MSW management scenarios representing mitigation and/or adaptation challenges. A short description of the narratives in terms of economic development and demographics for each SSPs along with the description of MSW management scenarios is presented below:

Table S6. MSW management narratives

**SSP1**	**"Sustainability Scenario"**
Socio-economic aspects	Economic growth is moderately high in developing countries leading to a reduction of inequality within and between countries
	Low material growth and resource use.
	Current high fertility countries move towards low population while in low fertility countries there is an increase of fertility rates. Urbanization is still high in developing countries, the negative effects associated with it are limited.
Environmental aspects	Environmental aspects are of high priority.
	Maximum municipal food waste reduction of 50% by the year 2030 based on Lipinski et al., 2013[19] and based on the target adopted by the United Nations Assembly in 2015 of halving per capita food waste at the retail and consumer level as a part of the 2030 Sustainable Development Goals.
	A maximum municipal plastic waste rate reduction of 50% by the year 2030 as a part of the 2030 Sustainable Development Goals.
Technological aspects	Waste technology transfer and capacity building is facilitated allowing the less favored countries to improve and develop appropriate waste management systems in both urban and rural areas. Hence, environmental impacts such as air pollution and GHGs emissions caused by inappropriate waste management are avoided.
Collection rates	2030: Increase global collection rates to 95% in rural areas and to 98% in urban areas.<br>2040: Increase global collection rates to > 99% in urban and rural areas.
Waste treatment	2030: 70% of total global food and garden waste treated in anaerobic digestion or (household) composting in rural areas.<br>2030: 55% of total global food and garden waste treated in anaerobic digestion or (largescale) composting in urban areas.<br>2040: ~98% of total global food and garden waste treated in anaerobic digestion or composting in urban and rural areas.<br><br>2030: Increase the global total recycling of materials up to 35% as follows: Plastic recycling ~70% in urban and rural areas, paper recycling up to ~70% in rural and ~80% in urban areas, glass recycling ~80% in rural and ~85% in urban areas, metal ~75% in rural and ~80% in urban areas and textile ~70% in urban and rural areas. Scattered will be reduce to 10% in rural areas and to 5% in urban areas.<br><br>2040: Increase the global total recycling of materials up to 45% as follows: Plastic recycling ~75% in urban and rural areas, paper recycling up to ~85% in rural and urban areas, glass recycling ~95% in rural and urban areas, metal ~85% in rural and urban areas and textile ~85% in urban and rural areas. Scattered will be reduce to < 1 % in rural and urban areas.

**SSP2**	**“Middle of the road”**
Socio-economic aspects	Moderate economic growth. Income distribution shows an improvement, but inequalities are still observed.
	Population growth is moderate, and urbanization is consistent with the historical trend.
Environmental aspects	Proactive or reactive depending on the region
	EU27 + UK, EU West, EU East, Oceania OECD, and North America regions continue developing and implementing policies to meet the proposed environmental targets related to waste.<br>Russia and the Former Soviet Union countries also implement similar policies but a slower pace.<br>All other countries either continue or start developing strategies to improve their waste management systems but are still left behind in terms of implementation.
Technological aspects	There is an improvement in the global waste management system at a global level, however, inequalities are observed in the developing countries. Although those countries start taking MSW waste management as an important point in the political agenda, the implementation of the MSW management strategies, although possible, is challenging. Technological capacity cannot keep pace with the quantities of MSW generation
Collection rates	2030: Increase global collection rates to 88% in rural areas and to 96% in urban areas.<br>2040: Increase global collection rates to 96% in rural areas and to 98% in urban areas.
Waste treatment	2030: ~35% of total global food and garden waste treated in anaerobic digestion or (household) composting in rural areas.<br>2030: ~15% of total global food and garden waste treated in anaerobic digestion or (largescale) composting in urban areas.<br>2040: ~75% and 60% of total global food and garden waste treated in anaerobic digestion or composting in rural and urban areas, respectively.<br><br>2030: Increase the global total recycling of materials up to 15% as follows: Plastic recycling ~25% in rural and urban areas, paper recycling up to ~45% in rural and urban areas, glass, and metal ~50% in urban and rural areas, and textile between 25% and 35% in urban and rural areas. Scattered waste will be reduced to 30% in rural areas and to 12% in urban areas.<br><br>2040: Increase the global total recycling of materials up to 35% as follows: Plastic recycling ~75% in rural and urban areas, paper recycling up to ~85% in rural and urban areas, glass, and metal recycling up to 85% in rural and urban areas, and textile ~70% in urban and rural areas. Scattered will be reduce to 10 % in rural and 5% in urban areas.

**SSP3**	**“Regional Rivalry”**
Socio-economic aspects	Slight economic growth due to lack of investment in education and technology. High inequalities within and between countries.
	Population growth is high, and urbanization slow.
Environmental aspects	Reactive to environmental problems.
	EU27+UK, EU West, EU East, Oceania OECD, and North America countries continue developing and implementing policies to meet the proposed environmental targets related to waste.<br>Russia and The Former Soviet Union countries also implement similar policies but a slower pace.<br>All other countries are behind in terms of policies and adoption of technologies but still show somehow an improvement with difficulties, especially in rural areas. Environmental concerns related to waste are not a priority in these countries.
Technological aspects	Absence of international support in terms of technology transfer and capacity building. Disparities between waste management in urban and rural areas.
Collection rates	2030: Increase global collection rates to 85% in rural areas and to 96% in urban areas.<br>2040: Increase global collection rates to 88% in rural areas and to 98% in urban areas.
Waste treatment	2030: ~30% of total global food and garden waste treated in anaerobic digestion or (household) composting in rural areas.<br>2030: ~10% of total global food and garden waste treated in anaerobic digestion or (largescale) composting in urban areas.<br>2040: ~55% of total global food and garden waste treated in anaerobic digestion or composting in rural and urban areas, respectively.<br><br>2030: Increase the global total recycling of materials up to 12% as follows: Plastic recycling ~20% in rural and urban areas, paper recycling up to ~40% in rural and urban areas, glass, and metal ~50% in urban and rural areas, and textile around 15% in urban and rural areas. Scattered waste will be reduced to ~35% in rural areas and to ~15% in urban areas.<br><br>2040: Increase the global total recycling of materials up to 30% as follows: Plastic recycling ~65% in rural and urban areas, paper recycling up to ~70% - 80% in rural and urban areas, glass, and metal recycling up to 70% - 80% in rural and urban areas, and textile ~65% in urban and rural areas. Scattered will be reduce to ~20 % in rural and 5% in urban areas.

**SSP4**	**“Inequality”**
Socio-economic aspects	Medium economic growth in high- and middle-income countries.
	Moderate population growth. Industrialized countries depict low fertility rates and population
Environmental aspects	Proactive in urban areas and reactive in rural
	EU27+UK, EU West, EU East, Oceania, and North America continue developing and implementing policies to meet the proposed environmental targets related to waste.<br>Russia and The Former Soviet Union countries catch up with European countries in terms of waste management.<br>All other countries continue struggling to cope with the large quantities of waste generated.
Technological aspects	Some technological development, especially in urban areas.
Collection rates	2030: Increase global collection rates to 87% in rural areas and to 96% in urban areas.<br>2040: Increase global collection rates to 90% in rural areas and to 98% in urban areas.
Waste treatment	2030: 35% of total global food and garden waste treated in anaerobic digestion or (household) composting in rural areas.<br>2030: 12% of total global food and garden waste treated in anaerobic digestion or (largescale) composting in urban areas.<br>2040: ~60% of total global food and garden waste treated in anaerobic digestion or composting in rural and urban areas, respectively.<br><br>2030: Increase the global total recycling of materials up to 15% as follows: Plastic recycling ~25% in rural and urban areas, paper recycling up to ~45% in rural and urban areas, glass, and metal ~45% in urban and rural areas, and textile around 15% in urban and rural areas. Scattered waste will be reduced to ~30% in rural areas and to 10% in urban areas.<br><br>2040: Increase the global total recycling of materials up to 35% as follows: Plastic recycling ~70% in rural and urban areas, paper recycling up to ~70% - 80% in rural and urban areas, glass, and metal recycling up to 70% - 80% in rural and urban areas, and textile ~65% in urban and rural areas. Scattered will be reduce to ~14 % in rural and 4% in urban areas.

SSP5	**"Fossil Fueled Development"**
Socio-economic aspects	Income inequality decreases within regions and per capita income increases at a global level.
	Global population declines. Regions reach high level of urbanization. High resource use and consumption.
Environmental aspects	Reactive environmental management as a response to unsustainable economy growth
	Waste technology transfer and capacity building is facilitated allowing the less favored countries to improve and develop appropriate waste management systems in both urban and rural areas. However, policies targeted to waste reduction are still missing.
Technological aspects	High potential for technological development (end-of-pipe solutions)
Collection rates	2030: Increase global collection rates to 95% in rural areas and to 98% in urban areas.<br>2040: Increase global collection rates to > 99% in urban and rural areas.
Waste treatment	2030: 70% of total global food and garden waste treated in anaerobic digestion or (household) composting in rural areas.<br>2030: 55% of total global food and garden waste treated in anaerobic digestion or (largescale) composting in urban areas.<br>2040: ~98% of total global food and garden waste treated in anaerobic digestion or composting in urban and rural areas.<br><br>2030: Increase the global total recycling of materials up to 33% as follows: Plastic recycling ~70% in urban and rural areas, paper recycling up to ~70% in rural and ~80% in urban areas, glass recycling ~80% in rural and ~85% in urban areas, metal ~75% in rural and ~80% in urban areas and textile ~65% in urban and rural areas. Scattered will be reduce to 10% in rural areas and to 5% in urban areas.<br><br>2040: Increase the global total recycling of materials up to 42% as follows: Plastic recycling ~75% in urban and rural areas, paper recycling up to ~85% in rural and urban areas, glass recycling ~92% in rural and urban areas, metal ~82% in rural and urban areas and textile ~83% in urban and rural areas. Scattered will be reduce to < 1 % in rural and urban areas.

Source: Based on Gómez-Sanabria et al., 2022[17] and further developed in this study.

S7. Development of MSW management by mitigation scenario and fraction of scattered MSW by stream

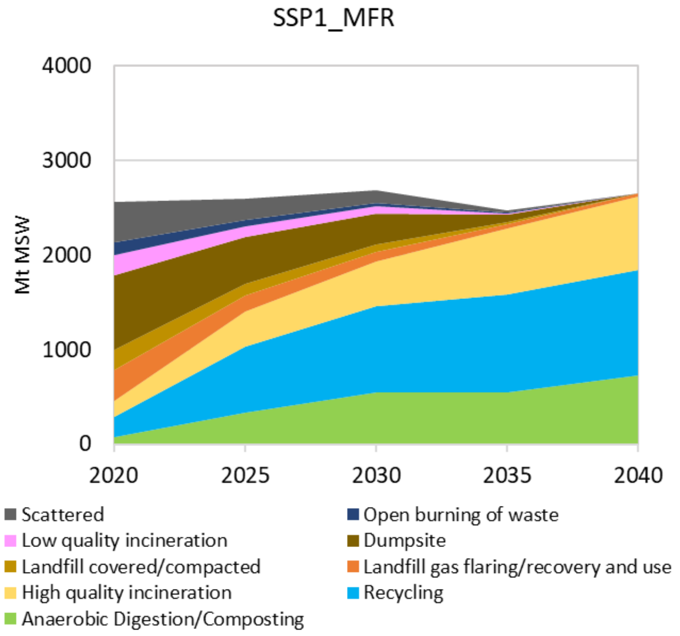

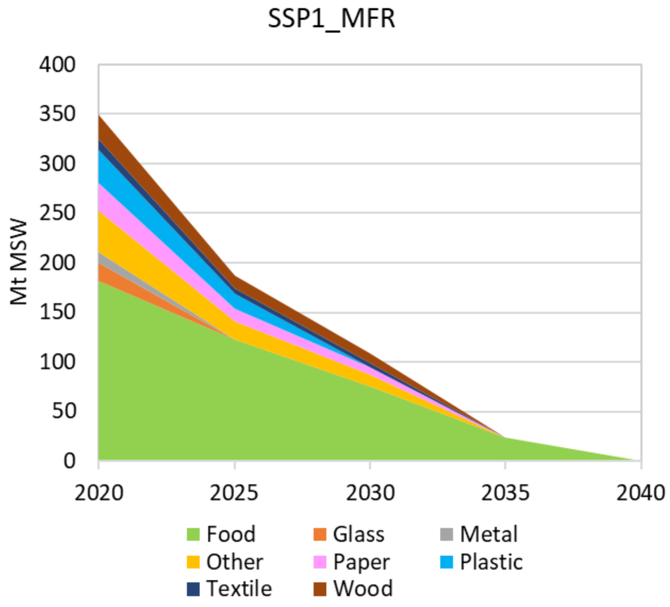

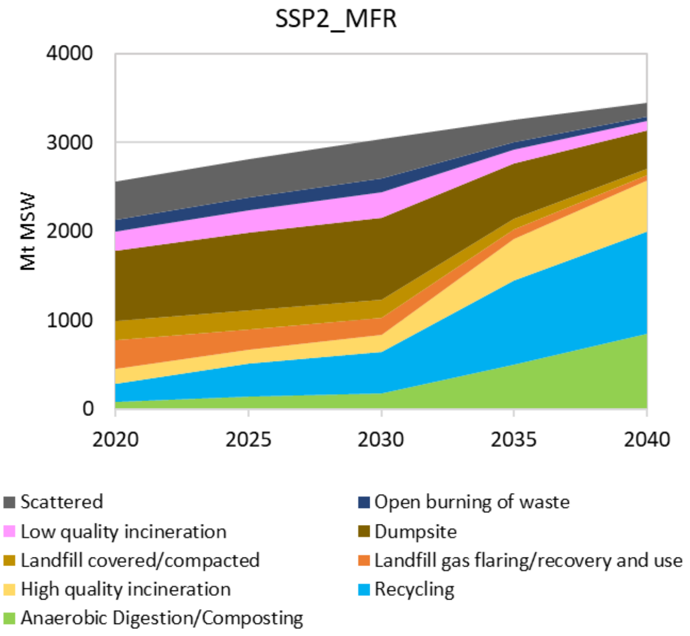

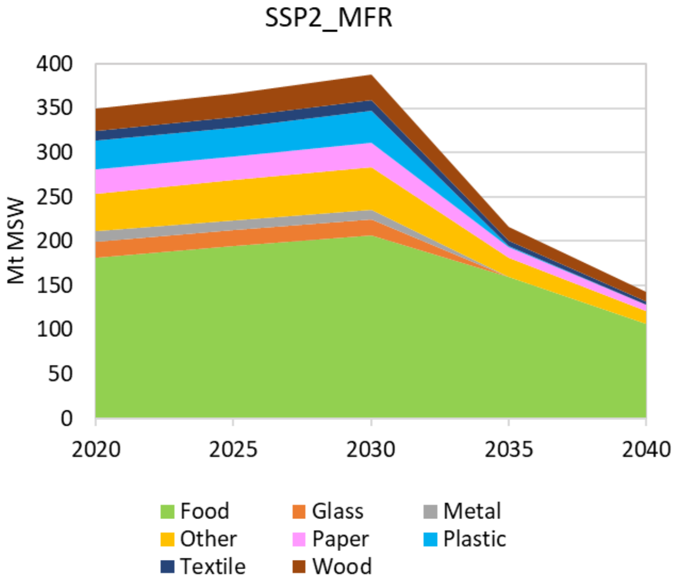

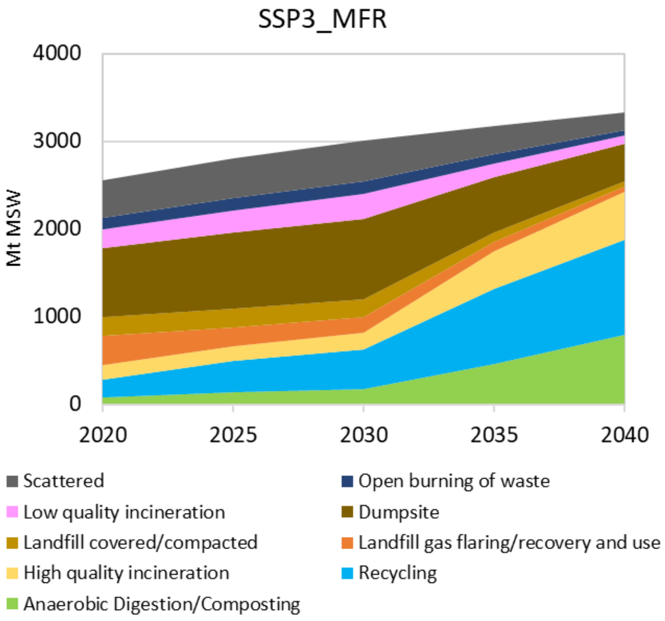
SSP3_MFR
Mt MSW
4000
3000
2000
1000
0
2020
2025
2030
2035
2040
Scattered
Open burning of waste
Low quality incineration
Dumpsite
Landfill covered/compacted
Landfill gas flaring/recovery and use
High quality incineration
Recycling
Anaerobic Digestion/Composting

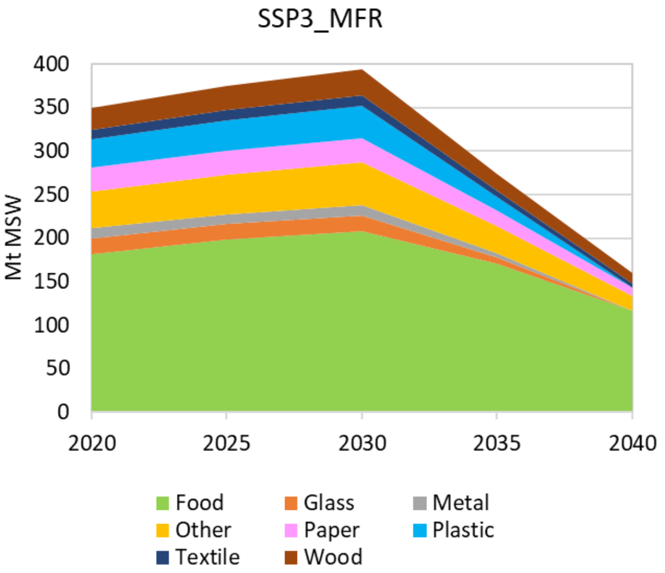
SSP3_MFR
Mt MSW
400
350
300
250
200
150
100
50
0
2020
2025
2030
2035
2040
Food
Glass
Metal
Other
Paper
Plastic
Textile
Wood

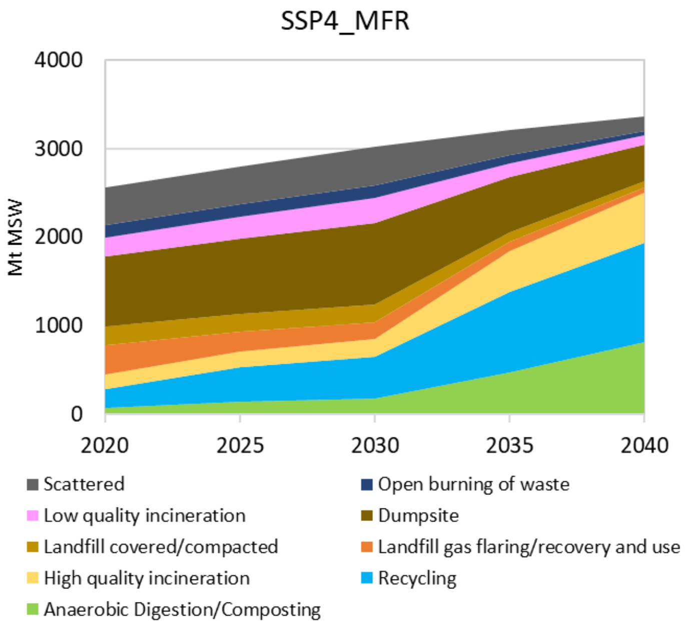
SSP4_MFR
Mt MSW
4000
3000
2000
1000
0
2020
2025
2030
2035
2040
Scattered
Open burning of waste
Low quality incineration
Dumpsite
Landfill covered/compacted
Landfill gas flaring/recovery and use
High quality incineration
Recycling
Anaerobic Digestion/Composting

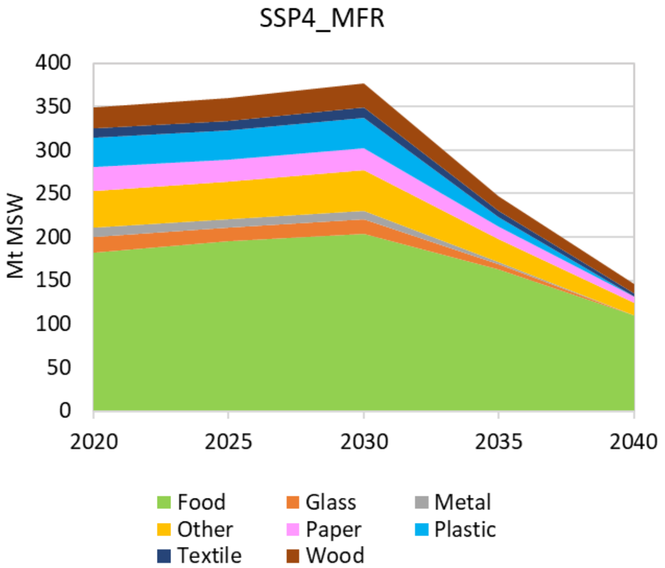
SSP4_MFR
Mt MSW
400
350
300
250
200
150
100
50
0
2020
2025
2030
2035
2040
Food
Glass
Metal
Other
Paper
Plastic
Textile
Wood

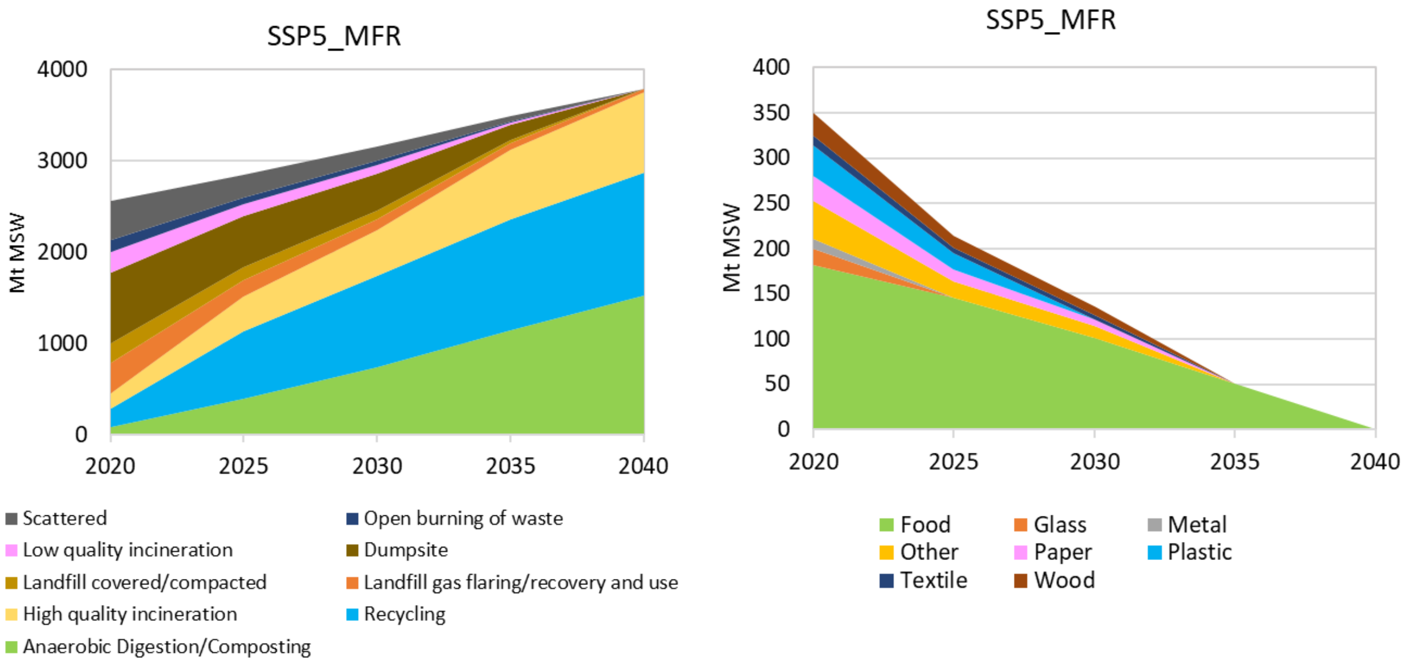

Fig S4. Development of MSW management in the mitigation scenarios and impact of scattered MSW by stream.

S8. GAINS country/region and regional aggregation

Table S7. GAINS country/region and regional aggregation

**Regional aggregation**	**Country/region in the GAINS model**
Africa	South Africa, Tanzania, Egypt, Kenya, Nigeria, North Africa (includes Algeria, Morocco, Libya, Tunisia, Sudan), East Africa, Western Africa, Rest Africa
China	Anhui, Beijing, Chongqing, Fujian, Gansu, Guangdong, Guangxi, Guizhou, Hainan, Hebei, Heilongjiang, Henan, Hong Kong and Macau, Hubei, Hunan, Jilin, Jiangsu, Jiangxi, Liaoning, Inner Mongolia, Ningxia, Qinghai, Shaanxi, Shanghai, Sichuan, Tianjin, Tibet, Xinjiang, Yunnan and Zhejiang
EU28	Austria, Belgium, Bulgaria, Cyprus, Croatia, Czech Republic, Denmark, Finland, France, Germany, Ireland, Luxembourg, Netherlands, Sweden, Greece, Malta, Portugal, Slovenia, Estonia, Hungary, Latvia, Lithuania, Poland, Romania, Slovak Republic, Italy, Spain, United Kingdom.
EU-East	Albania, Bosnia and Herzegovina, Kosovo, Macedonia, Montenegro, Serbia, Turkey.
EU-West	Norway, Iceland, Switzerland
Former Soviet Union (FSOV)	Armenia, Former Soviet Union States (includes Tajikistan, Turkmenistan, and Uzbekistan), Georgia, Azerbaijan, Kazakhstan, Belarus, Moldova, Kyrgyzstan
India	Andhra Pradesh, Assam, West Bengal, Bihar, Chhattisgarh, Delhi, North East (excl Assam), Goa, Gujarat, Haryana, Himachal Pradesh, Jharkhand, Karnataka, Kerala, Maharashtra, Manipur, Orissa, Punjab, Rajasthan, Tamil Nadu, Uttarakhand, Uttar Pradesh, Jammu Kashmir
Latin America and The Caribbean	Argentina, Caribbean (includes countries in the Caribbean region), Chile, Brazil, Mexico, Central America, Colombia, Ecuador, Bolivia, Paraguay, Perú, Uruguay, Venezuela, and Other Latin America.
Middle East	Middle East, Iran, Israel, Saudi Arabia
North America	United States and Canada.
Oceania OECD	Australia, New Zealand, Japan
Russia	Russia (Europe – Asia)
South Asia	Afghanistan, Bangladesh (Dhaka and rest of Bangladesh), Cambodia, North Korea, South Korea, Myanmar, Taiwan, Nepal, Pakistan (Karachi, NW frontier provinces Baluchistan, Punjab and Sindh), Philippines (Bicol, Luzol and Manila), Sri Lanka, Thailand (Bangkok, Central Valley, North Eastern Plateau, Northern Highlands and Southern Peninsula), Vietnam (North and South).

Source: Gómez-Sanabria et al., 2022[17].

S9. Sensitivity

Table S8. Average Coefficient of Variation of the sensitivity analysis based on 100 samples

Variable	CV (100 Samples)
Uncollected waste	1.690
Fate of Leakage	1.246
Population up to 1 km	1.182

References

1. Lebreton, L. C. M. *et al.* River plastic emissions to the world's oceans. *Nature Communications* **8**, 15611 (2017).
2. Meijer, L. J. J., van Emmerik, T., van der Ent, R., Schmidt, C. & Lebreton, L. More than 1000 rivers account for 80% of global riverine plastic emissions into the ocean. *Science Advances* **7**, eaaz5803 (2019).
3. Lebreton, L. & Andrady, A. Future scenarios of global plastic waste generation and disposal. *Palgrave Communications* **5**, 6 (2019).
4. Schmidt, C., Krauth, T. & Wagner, S. Export of Plastic Debris by Rivers into the Sea. *Environ. Sci. Technol.* **51**, 12246–12253 (2017).
5. Jambeck, J. R. *et al.* Marine pollution. Plastic waste inputs from land into the ocean. *Science* **347**, 768–771 (2015).
6. OECD. *Global Plastics Outlook: Economic Drivers, Environmental Impacts and Policy Options,*. https://doi.org/10.1787/de747aef-en (2022).
7. UNEP. *From Pollution to Solution: A global assessment of marine litter and plastic pollution. Synthesis.* (2021).
8. Lau, W. W. Y. *et al.* Evaluating scenarios toward zero plastic pollution. *Science* **369**, 1455–1461 (2020).
9. Borrelle, S. B. *et al.* Predicted growth in plastic waste exceeds efforts to mitigate plastic pollution. *Science* **369**, 1515–1518 (2020).
10. RWA. *Plastic Waste Management in Lake Hawassa Basin*. (2020).
11. Hoffman, M. J. & Hittinger, E. Inventory and transport of plastic debris in the Laurentian Great Lakes. *Marine Pollution Bulletin* **115**, 273–281 (2017).
12. Mihai, F.-C. Rural plastic emissions into the largest mountain lake of the Eastern Carpathians. *R Soc Open Sci* **5**, 172396 (2018).
13. Liro, M. *et al.* Mountains of plastic: Mismanaged plastic waste along the Carpathian watercourses. *Science of The Total Environment* **888**, 164058 (2023).
14. Rinasti, A. N., Ibrahim, I. F., Gunasekara, K., Koottatep, T. & Winijkul, E. Fate identification and management strategies of non-recyclable plastic waste through the integration of material flow analysis and leakage hotspot modeling. *Scientific Reports* **12**, 16298 (2022).
15. Van Emmerik, T., Loozen, M., Van Oeveren, K., Buschman, F. & Prinsen, G. Riverine plastic emission from Jakarta into the ocean. *Environmental Research Letters* **14**, 084033 (2019).
16. Winterstetter, A., Mira Veiga, J., Sholokhova, A. & Šubelj, G. Country-specific assessment of mismanaged plastic packaging waste as a main contributor to marine litter in Europe. *Frontiers in Sustainability* **3**, (2023).
17. Gómez-Sanabria, A., Kiesewetter, G., Klimont, Z., Schoepp, W. & Haberl, H. Potential for future reductions of global GHG and air pollutants from circular waste management systems. *Nature Communications* **13**, 106 (2022).
18. The European Union Parlament and the Council. Directive (EU) 2018/851 of the European Parliament and of the Council of 30 May 2018 amending Directive 2008/98/EC on waste. (2018).
19. O'Neill, B. C. *et al.* The roads ahead: Narratives for shared socioeconomic pathways describing world futures in the 21st century. *Global Environmental Change* **42**, 169–180 (2017).
