# Supplementary material for: The crucial role of circular waste management systems in cutting waste leakage into aquatic environments
Source: Nat Commun. 2024 Jun 27;15:5443. doi: 10.1038/s41467-024-49555-9 (PMC11211435; doi:10.1038/s41467-024-49555-9)
Supplement: Supplementary file 3 — Description of Additional Supplementary Files [file 41467_2024_49555_MOESM3_ESM.pdf]

Description of Additional Supplementary Files

File Name: Supplementary Data 1
Description: File providing detailed data complementing the manuscript.
