# Supplementary material for: The crucial role of circular waste management systems in cutting waste leakage into aquatic environments
Source: Nat Commun. 2024 Jun 27;15:5443. doi: 10.1038/s41467-024-49555-9 (PMC11211435; doi:10.1038/s41467-024-49555-9)
Supplement: Supplementary file 5 — Reporting Summary [file 41467_2024_49555_MOESM5_ESM.pdf]

nature portfolio

Corresponding author(s): Gomez-Sanabria Adriana
NCOMMS-23-14978C

Last updated by author(s): Apr 30, 2024

Reporting Summary

Nature Portfolio wishes to improve the reproducibility of the work that we publish. This form provides structure for consistency and transparency in reporting. For further information on Nature Portfolio policies, see our Editorial Policies and the Editorial Policy Checklist.

Statistics

For all statistical analyses, confirm that the following items are present in the figure legend, table legend, main text, or Methods section.

n/a	Confirmed	
x		The exact sample size (*n*) for each experimental group/condition, given as a discrete number and unit of measurement
x		A statement on whether measurements were taken from distinct samples or whether the same sample was measured repeatedly
	x	The statistical test(s) used AND whether they are one- or two-sided<br>*Only common tests should be described solely by name; describe more complex techniques in the Methods section.*
x		A description of all covariates tested
x		A description of any assumptions or corrections, such as tests of normality and adjustment for multiple comparisons
	x	A full description of the statistical parameters including central tendency (e.g. means) or other basic estimates (e.g. regression coefficient) AND variation (e.g. standard deviation) or associated estimates of uncertainty (e.g. confidence intervals)
	x	For null hypothesis testing, the test statistic (e.g. *F*, *t*, *r*) with confidence intervals, effect sizes, degrees of freedom and *P* value noted<br>*Give P values as exact values whenever suitable.*
x		For Bayesian analysis, information on the choice of priors and Markov chain Monte Carlo settings
x		For hierarchical and complex designs, identification of the appropriate level for tests and full reporting of outcomes
x		Estimates of effect sizes (e.g. Cohen's *d*, Pearson's *r*), indicating how they were calculated

Our web collection on statistics for biologists contains articles on many of the points above.

Software and code

Policy information about availability of computer code

Data collection	The data were processed in R version 4.0.4, Microsoft Excel, PostGIS, PostreSQL, ArcGIS
Data analysis	The data were processed in R version 4.0.4, Microsoft Excel, PostGIS, PostreSQL, ArcGIS

For manuscripts utilizing custom algorithms or software that are central to the research but not yet described in published literature, software must be made available to editors and reviewers. We strongly encourage code deposition in a community repository (e.g. GitHub). See the Nature Portfolio guidelines for submitting code & software for further information.

Data

Policy information about availability of data

All manuscripts must include a data availability statement. This statement should provide the following information, where applicable:

- Accession codes, unique identifiers, or web links for publicly available datasets
- A description of any restrictions on data availability
- For clinical datasets or third party data, please ensure that the statement adheres to our policy

All data generated during this study is included in this published article (and its Supplementary Information). The Supplementary Data generated in this study has been deposited in https://doi.org/10.6084/m9.figshare.23855370. Source data are provided with this paper.
Databases used: SSP Public Database version 2.0. Population, GDP and Urbanization data. https://tntcat.iiasa.ac.at/SspDb/dsd?Action=htmlpage&page=about
GHSL-Global Human Settlement Layer Population Count: 10.2905/D6D86A90-4351-4508-99C1-CB074B022C4A

GHSL-Global Human Settlement Layer Degree of Urbanisation: 10.2905/4606D58A-DC08-463C-86A9-D49EF461C47F
FAO (Administrative Boundaries and Coastlines): https://data.apps.fao.org/map/catalog/srv/eng/catalog.search#/metadata/9c35ba10-5649-41c8-bdfc-eb78e9e65654
http://cidportal.jrc.ec.europa.eu/ftp/jrc-opendata/GHSL/GHS_STAT_UCDB2015MT_GLOBE_R2019A/V1-2/
HydroLakes: https://www.hydrosheds.org/products/hydrolakes / https://doi.org/10.1002/hyp.9740
HydroRivers: https://www.hydrosheds.org/products/hydrorivers
PostGIS,PostGIS; http://postgis.net/
PostreSQL,PostgreSQL; http://www.postgresql.org/

Research involving human participants, their data, or biological material

Policy information about studies with human participants or human data. See also policy information about sex, gender (identity/presentation), and sexual orientation and race, ethnicity and racism.

Reporting on sex and gender	*Use the terms sex (biological attribute) and gender (shaped by social and cultural circumstances) carefully in order to avoid confusing both terms. Indicate if findings apply to only one sex or gender; describe whether sex and gender were considered in study design; whether sex and/or gender was determined based on self-reporting or assigned and methods used.*<br>*Provide in the source data disaggregated sex and gender data, where this information has been collected, and if consent has been obtained for sharing of individual-level data; provide overall numbers in this Reporting Summary. Please state if this information has not been collected.*<br>*Report sex- and gender-based analyses where performed, justify reasons for lack of sex- and gender-based analysis.*
Reporting on race, ethnicity, or other socially relevant groupings	*Please specify the socially constructed or socially relevant categorization variable(s) used in your manuscript and explain why they were used. Please note that such variables should not be used as proxies for other socially constructed/relevant variables (for example, race or ethnicity should not be used as a proxy for socioeconomic status).*<br>*Provide clear definitions of the relevant terms used, how they were provided (by the participants/respondents, the researchers, or third parties), and the method(s) used to classify people into the different categories (e.g. self-report, census or administrative data, social media data, etc.)*<br>*Please provide details about how you controlled for confounding variables in your analyses.*
Population characteristics	*Describe the covariate-relevant population characteristics of the human research participants (e.g. age, genotypic information, past and current diagnosis and treatment categories). If you filled out the behavioural & social sciences study design questions and have nothing to add here, write "See above."*
Recruitment	*Describe how participants were recruited. Outline any potential self-selection bias or other biases that may be present and how these are likely to impact results.*
Ethics oversight	*Identify the organization(s) that approved the study protocol.*

Note that full information on the approval of the study protocol must also be provided in the manuscript.

Field-specific reporting

Please select the one below that is the best fit for your research. If you are not sure, read the appropriate sections before making your selection.

☐ Life sciences ☐ Behavioural & social sciences ☒ Ecological, evolutionary & environmental sciences

For a reference copy of the document with all sections, see nature.com/documents/nr-reporting-summary-flat.pdf

Ecological, evolutionary & environmental sciences study design

All studies must disclose on these points even when the disclosure is negative.

Study description	By contrasting baseline with mitigation scenarios, our study combines spatial analysis with the Shared Socioeconomic Pathways storylines to develop plausible future waste leakage mitigation strategies up to 2040 resulting from the implementation of circular MSW management systems.
Research sample	The selection of the databases used in this study are based on the representativeness and recognition at a global level. For example, the SSPs are an important input for the recent and ongoing IPCC Assessment Reports and are central to the climate research community, GHSL-Global Human Settlement Layer Population Count and GHSL-Global Human Settlement Layer Degree of Urbanisation from JRC are globally recognized as a good representation of population and population distribution. The administrative boundaries (GAUL) from FAO compiles and disseminates the best available information on administrative units for all the countries in the world, HydroLakes and HydroRivers are databases with global representation of aquatic environments widely recognized.
Sampling strategy	The databases were selected based on the recognition in the scientific community.
Data collection	The datasets were directly downloaded from the corresponding repositories.
Timing and spatial scale	The study has global coverage with a geographic representation of 180 country/regions with multi temporal resolution at five years

	intervals. The study differentiates between urban and rural areas within a country/region and types of MSW management by MSW fraction. The spatial data is represented by main river with a classical stream order higher than three and lakes > 50km2
Data exclusions	Rivers with a classical stream order lower than three and lakes with an area smaller than 50 km2
Reproducibility	All data generated during this study is available at GAINS country/regional level, including urban and rural areas, MSW fractions (i.e., food, paper, plastic, glass, metal, etc), and Scenario. All attempts at replication of data were successful.
Randomization	This is not relevant to our study
Blinding	This is not relevant to our study

Did the study involve field work? ☐ Yes ☒ No

Reporting for specific materials, systems and methods

We require information from authors about some types of materials, experimental systems and methods used in many studies. Here, indicate whether each material, system or method listed is relevant to your study. If you are not sure if a list item applies to your research, read the appropriate section before selecting a response.

Materials & experimental systems

n/a	Involved in the study
☒	☐ Antibodies
☒	☐ Eukaryotic cell lines
☒	☐ Palaeontology and archaeology
☒	☐ Animals and other organisms
☒	☐ Clinical data
☒	☐ Dual use research of concern
☒	☐ Plants

Methods

n/a	Involved in the study
☒	☐ ChIP-seq
☒	☐ Flow cytometry
☒	☐ MRI-based neuroimaging
